# Nomogram incorporating preoperative clinical and ultrasound indicators to predict aggressiveness of solitary papillary thyroid carcinoma

**DOI:** 10.3389/fonc.2023.1009958

**Published:** 2023-01-31

**Authors:** Long Liu, Chao Jia, Gang Li, Qiusheng Shi, Lianfang Du, Rong Wu

**Affiliations:** ^1^ Department of Ultrasound, Shanghai General Hospital of Nanjing Medical University, Shanghai, China; ^2^ Department of Ultrasound, Shanghai General Hospital, Shanghai Jiao Tong University School of Medicine, Shanghai, China

**Keywords:** papillary thyroid carcinoma, thyroid, ultrasound, nomogram, aggressiveness

## Abstract

**Objective:**

To construct a nomogram based on preoperative clinical and ultrasound indicators to predict aggressiveness of solitary papillary thyroid carcinoma (PTC).

**Methods:**

Preoperative clinical and ultrasound data from 709 patients diagnosed with solitary PTC between January 2017 and December 2020 were analyzed retrospectively. Univariate and multivariate logistic regression analyses were performed to identify the factors associated with PTC aggressiveness, and these factors were used to construct a predictive nomogram. The nomogram’s performance was evaluated in the primary and validation cohorts.

**Results:**

The 709 patients were separated into a primary cohort (n = 424) and a validation cohort (n = 285). Univariate analysis in the primary cohort showed 13 variables to be associated with aggressive PTC. In multivariate logistic regression analysis, the independent predictors of aggressive behavior were age (OR, 2.08; 95% CI, 1.30-3.35), tumor size (OR, 4.0; 95% CI, 2.17-7.37), capsule abutment (OR, 2.53; 95% CI, 1.50-4.26), and suspected cervical lymph nodes metastasis (OR, 2.50; 95% CI, 1.20-5.21). The nomogram incorporating these four predictors showed good discrimination and calibration in both the primary cohort (area under the curve, 0.77; 95% CI, 0.72-0.81; *Hosmer*–Lemeshow *test*, *P* = 0.967 and the validation cohort (area under the curve, 0.72; 95% CI, 0.66-0.78; *Hosmer*–Lemeshow *test*, *P* = 0.251).

**Conclusion:**

The proposed nomogram shows good ability to predict PTC aggressiveness and could be useful during treatment decision making.

**Advances in knowledge:**

Our nomogram—based on four indicators—provides comprehensive assessment of aggressive behavior of PTC and could be a useful tool in the clinic.

## Introduction

Papillary thyroid cancer (PTC) accounts for 80%-85% of follicular cell–derived thyroid cancers (1). It generally follows an indolent course, with five-year survival rate >90% (2, 3), but may sometimes show aggressive behavior and present with capsular invasion (CAI), extrathyroidal extension (ETE), vascular invasion, and regional and distant metastasis (DM) (4–9). Early awareness of aggressive behavior can help clinicians select the appropriate management approach (10) but, unfortunately, the aggressive nature of the tumor is usually recognized only after surgery. It is therefore important to develop methods for preoperative prediction of aggressiveness.

Preoperative computed tomography and magnetic resonance imaging can display ETE and extent of invasion of larynx, trachea, and esophagus (11), but these modalities have disadvantages. Computed tomography exposes the patient to ionizing radiation and is not as accurate as ultrasonography for judging ETE or diagnosing lateral cervical lymph node metastasis (CLNM) (12). Magnetic resonance imaging can assess the spread of PTC but is susceptible to respiratory movement artifacts and is not as sensitive as ultrasonography for diagnosis of lymph node metastasis (13–16). Ultrasonography, the most commonly used imaging modality for preoperative differentiation of benign from malignant thyroid nodules (11, 17, 18), is useful for determining the extent of CAI, ETE, and CLNM (19–21), but conventional ultrasound (CUS) may not be able to show CAI and ETE when the nodule is situated close to the posterior thyroid capsule. CUS has high accuracy in the diagnosis of lateral CLNM, but it has poor sensitivity (only 30%) for central CLNM (due to intervening bone and gas) (22). Contrast-enhanced ultrasound (CEUS) has been shown to be more accurate than CUS for judging ETE and CLNM (23–25).

The aim of this study was to identify the clinical and ultrasonographic indicators of aggressive behavior of PTC and to use them to construct a nomogram for preoperative prediction of aggressiveness of solitary PTC.

## Materials and methods

This retrospective study was performed in accordance with the principles outlined in the Declaration of Helsinki and was approved by the Institutional Review Board of our Hospital. The need for informed consent was waived as this was a retrospective analysis of anonymized data.

### Patients

**Figure 1:**
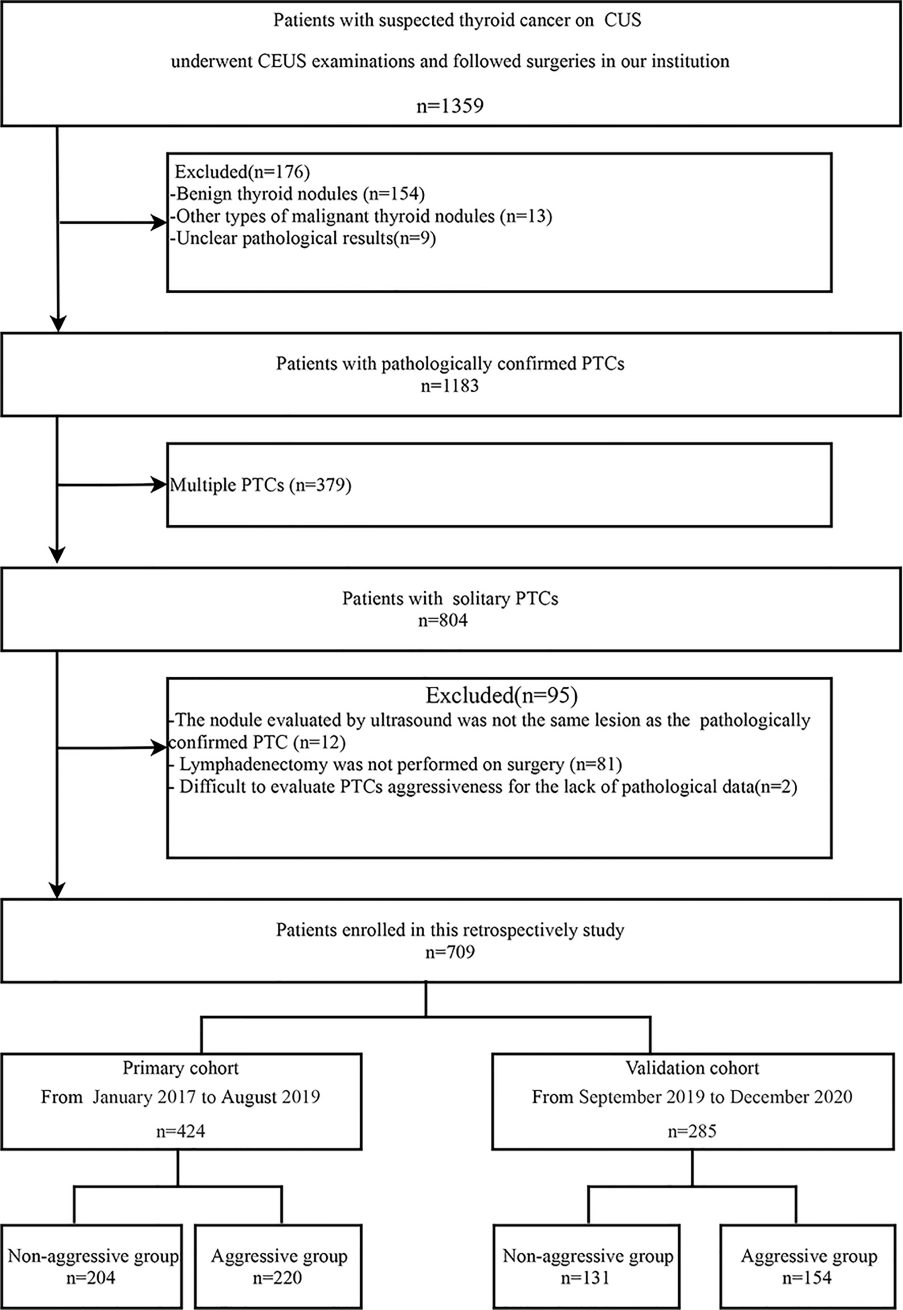
Flow chart showing patient selection process. PTC, papillary thyroid carcinoma; CUS, conventional ultrasound; CEUS, contrast enhanced ultrasound.

We reviewed the medical records of 1359 patients who underwent surgeries for suspected thyroid cancer based on preoperative fine needle aspiration cytology findings at our hospital between January 2017 and December 2020. All patients underwent CUS and CEUS examinations before surgery. A total of 709 patients with solitary PTC who met the eligibility criteria were selected for the study. The flowchart in **Figure 1** shows the inclusion and exclusion criteria and the patient selection process.

### Ultrasound examination

Ultrasound examinations were performed using an Aplio 500 (TOSHIBA, Japan) system equipped with a 7-14MHz linear probe and an Aplio i900 (Canon, Japan) system equipped with a 5-18MHZ probe. CEUS was performed using a low mechanical index mode (mechanical indices were 0.12 in the Aplio 500 and 0.04 in the Aplio i900).

On CUS, the following features of PTCs were evaluated: 1) size (≤10 mm or >10 mm); 2) margin (well defined or poorly defined); 3) shape (regular or irregular); 4) aspect ratio (height divided by width on transverse views); 5) calcification (no calcification, microcalcification, macrocalcification, or mixed micro- and macrocalcification); 6) capsule abutment (CA; defined as tumor located adjacent to the capsule with no intervening normal thyroid tissue); 7) loss of echogenic capsule (defined as absence of the normally detected echogenic rim of the thyroid); 8) suspected cervical lymph node metastasis (SCLNM; indicated by loss of the fatty hilum, peripheral vascular flow on color Doppler imaging, increased cortical echogenicity, and a rounded shape [long axis to short axis ratio of <1.5], cystic changes, and microcalcification); and 9) tumor vascularity (classified as types I, II, III, or IV (26)).

On CEUS images, the following features were evaluated: 1) time to tumor enhancement, which was classified as early enhancement (i.e., tumor enhancement before enhancement of surrounding thyroid parenchyma), late enhancement (tumor enhancement after enhancement of surrounding thyroid parenchyma), or simultaneous enhancement (simultaneous enhancement of tumor and surrounding thyroid parenchyma); 2) enhancement intensity of tumor relative to enhancement intensity of surrounding thyroid parenchyma at peak enhancement (classified as hyper-enhancement, iso-enhancement, or hypo-enhancement); 3) homogeneity of tumor enhancement (homogeneous or heterogeneous); 4) discontinuous capsule enhancement (discontinuity in the hyperechoic thyroid capsule).

### Image analysis

Two radiologists (each with more than 5 years’ experience in thyroid US and CEUS) blinded to the pathological results evaluated the CUS and CEUS images independently; both radiologists were not otherwise involved in this study. Discrepancies were resolved by consensus or by consultation with a senior radiologist (with more than 20 years’ experience in thyroid CUS and 10 years’ experience in thyroid CEUS). Inter-reader agreement was assessed.

### Pathological evaluation

The tumors were classified as aggressive or non-aggressive based on pathologic findings, radiological results, or nuclear imaging findings. Aggressive PTCs were those with one or more of the following: CAI, ETE, lymphatic or vascular invasion, CLNM, and distant metastases. Non-aggressive tumors were those with none of the above features. Pathological indicators were evaluated by pathologists with 10 years of experience in diagnosis of thyroid pathology.

### Risk factor identification and nomogram development

First, in the primary cohort, univariate analysis was used to identify the variables significantly associated (at *P* < 0.05) with aggressive behavior. The identified variables were then entered into multivariable binary logistic regression analysis to identify those independently associated with aggressive behavior. The identified independent risk factors were used to construct the predictive nomogram.

### Statistical analysis

Statistical analysis was performed with SPSS 23.0 (IBM Corp., Armonk, NY, USA) and the *rms*, *pROC*, and *ResourceSelection* statistical packages in R 4.1.0 (The R Foundation for Statistical Computing, Vienna, Austria). Quantitative data were expressed as means ± standard deviation or medians (with upper and lower quartiles) according to the normality of the distribution and compared between groups using the *t* test or the nonparametric Mann–Whitney *U* test, respectively. Categorical data were summarized as percentages and compared between groups using the Pearson chi-square test or the Fisher exact test. Cohen’s *κ* analysis was used to evaluate inter-reader agreement.

The performance of the nomogram was assessed by evaluating discrimination and calibration. Receiver operating characteristic curve (ROC) analysis was used to evaluate the discrimination performance of the nomogram, and the area under the curve (AUC) was calculated. Calibration curves were plotted *via* bootstrapping with 1000 resamples. Finally, the performance of the model was tested in the validation cohort. The *Hosmer*–Lemeshow *test* was used to evaluate the goodness of fit of the nomogram. The variance inflation factor was used to evaluate the collinearity of variables included in the nomogram. A variance inflation factors greater than 10 indicates multicollinearity. All statistical tests were two-tailed, and *P* < 0.05 was considered to indicate a statistically significant difference. The code for model development and evaluation is displayed in the Appendix.

## Results

### Patient characteristics

A total of 709 patients were included in the study: 180 men (mean age, 42.7 years; age range, 18-80 years) and 529 women (mean age, 44.4 years; age range, 19-79 years). While 424 patients formed the primary cohort, the other 285 patients formed the validation cohort. In the primary cohort, there were 204 patients in the non-aggressive group and 220 in the aggressive group. In the validation cohort, there were 131 patients in the non-aggressive group and 154 patients in the aggressive group. **Table 1** shows the baseline characteristics of the patients.

**Table 1:** Baseline characteristics of patients in primary and validation cohorts.

	Primary Cohort (n = 424)			Validation Cohort (n = 285)		
Characteristic	Non-aggressive (n = 204)	Aggressive (n = 220)	*P*	Non-aggressive (n = 131)	Aggressive (n = 154)	*P*
Age			0.001			0.015
<45 y	83 (40.7)	126 (57.3)		62 (47.3)	95 (61.7)	
≥45 y	121 (59.3)	94 (42.7)		69 (52.7)	59 (38.3)	
Sex			0.162			0.057
Male	42 (20.6)	58 (26.4)		30 (22.9)	51 (33.1)	
Female	162 (79.4)	162 (73.6)		101 (77.1)	103 (66.9)	
Size ≤10 mm >10 mm	179 (87.7) 25 (12.3)	114 (51.8) 106 (48.2)	<0.001	112 (85.5) 19 (14.5)	98 (63.6) 56 (36.4)	<0.001
Location			0.531			0.478
Right lobe	95 (46.6)	109 (49.5)		57 (43.5)	78 (50.6)	
Left lobe	102 (50.0)	100 (45.5)		71 (54.2)	73 (47.4)	
Isthmus	7 (3.4)	11 (5.0)		3 (2.3)	3 (1.9)	
Hashimoto thyroiditis			0.860			0.222
Yes	55 (27.0)	61 (27.7)		39 (29.8)	36 (23.4)	
No	149 (73.0)	159 (72.3)		92 (70.2)	118 (76.6)	

Data are n (%).

Differences between groups were compared using the chi-square test.

In both the primary cohort and the validation cohort, patients in the aggressive group tended to be younger and to have larger tumor size than patients in the non-aggressive group (all *P* < 0.05). Sex ratio, tumor location, and prevalence of Hashimoto thyroiditis were comparable between the aggressive group and the non-aggressive group in both the primary cohort and the validation cohort (all *P* > 0.05).

### Ultrasound features in the primary cohort

In the primary cohort, tumor shape was not significantly different between the non-aggressive group and the aggressive group (*P* = 0.125), but all other indices (i.e., margin, aspect ratio, calcification, CA, loss of echogenic capsule, SCLNM, vascularity, time to enhancement, intensity of enhancement, homogeneity of enhancement, and discontinuous capsule enhancement) were significantly different between the two groups (all *P* < 0.05; **Table 2**).

**Table 2:** Comparison of ultrasound features between the non-aggressive group and aggressive group in the primary cohort.

Characteristics	Non-aggressive group (n = 204)	Aggressive group (n = 220)	χ^2^, Z	*P*
Margin			5.927	0.015
Well defined	46 (22.5)	73 (33.2)		
Poorly defined	158 (77.5)	147 (66.8)		
Shape			2.354	0.125
Regular	59 (28.9)	79 (35.9)		
Irregular	145 (71.1)	141 (64.1)		
Aspect ratio^a^	1.15 (1,1.33)	1.09 (0.89,1.28)	-2.879^b^	0.004
Vascularity			11.819	0.008
Type I	96 (47.1)	69 (31.4)		
Type II	12 (5.9)	22 (10.0)		
Type III	74 (36.3)	96 (43.6)		
Type IV	22 (10.8)	33 (15.0)		
Calcification			18.862	<0.001
No	115 (56.4)	91 (41.4)		
Microcalcification	60 (29.4)	99 (45.0)		
Macrocalcification	24 (11.8)	15 (6.8)		
Mixed mode	5 (2.5)	15 (6.8)		
CA			43.819	<0.001
No	101 (49.5)	42 (19.1)		
Yes	103 (50.5)	178 (80.9)		
Loss of echogenic capsule			7.453	0.006
No	191 (93.6)	188 (85.5)		
Yes	13 (6.4)	32 (14.5)		
SCLNM			10.995	0.001
No	189 (92.6)	180 (81.8)		
Yes	15 (7.4)	40 (18.2)		
Time to enhancement			6.688	00.035
Early	8 (3.9)	23 (10.5)		
Late	93 (45.6)	92 (41.8)		
Simultaneous	103 (50.5)	105 (47.7)		
Enhancement intensity			13.977	0.001
Hyper-enhancement	9 (4.4)	25 (11.4)		
Iso-enhancement	99 (48.5)	73 (33.2)		
Hypo-enhancement	96 (47.1)	122 (55.5)		
Homogeneity of enhancement			10.162	0.001
Homogeneous	127 (62.3)	103 (46.8)		
Heterogeneous	77 (37.7)	117 (53.2)		
Discontinuous capsule enhancement			12.551	<0.001
No	164 (80.4)	143 (65.0)		
Yes	40 (19.6)	77 (35.0)		

Data are n (%) unless otherwise indicated.

a Non-normally distributed data are summarized as the median (upper and lower quartiles).

Differences between groups were compared using the chi-square test unless specified.

b Differences between groups were compared using the nonparametric Mann–Whitney U test.

CA, capsule abutment; SCLNM, suspected cervical lymph node metastasis.

### Multivariate logistic regression analysis and development of the nomogram

**Figure 2:**
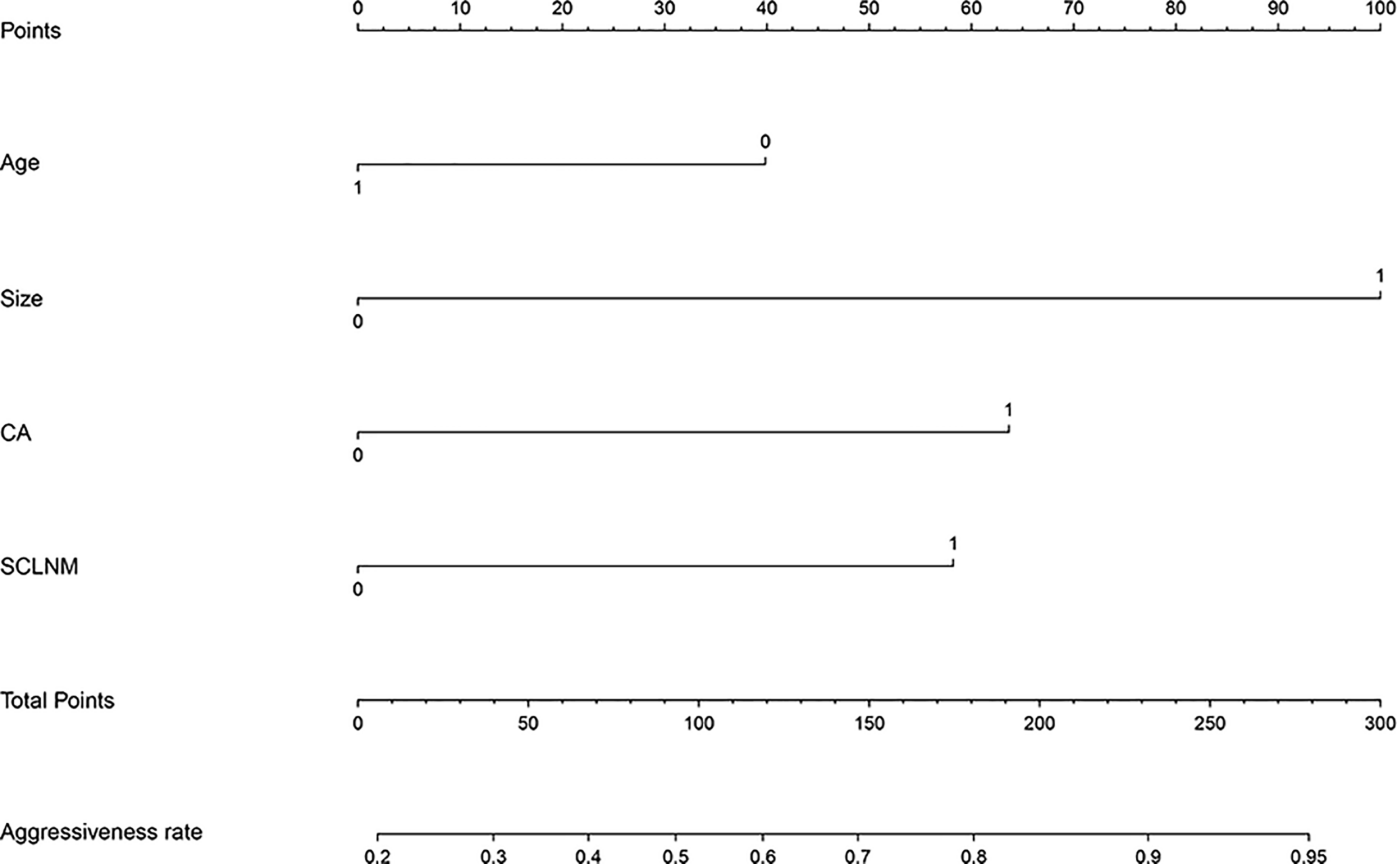
Nomogram to predict aggressiveness of papillary thyroid carcinoma (PTC). The nomogram incorporated patient age, tumor size, capsule abutment (CA), and suspected cervical lymph node metastasis (SCLNM).

**Table 3** presents the results of multivariate logistic regression analysis. Age <45 years (OR, 2.08; 95% CI, 1.30-3.35), tumor size > 10mm (OR, 4.00; 95% CI, 2.17-7.37), CA (OR, 2.53, 95% CI, 1.50-4.26), and SCLNM (OR, 2.50; 95% CI, 1.20-5.21) were the variables independently associated with PTC aggressiveness in the primary cohort (all *P* < 0.05). These four variables were used to construct the predictive nomogram (**Figure 2**).

**Table 3:** Multivariable logistic regression analysis in the primary cohort.

Variables	Adjusted OR	95%CI	*P*
Age			0.002
<45 years	2.08	1.30,3.35	
≥45 years	Ref.		
Size			<0.001
≤10 mm	Ref.		
>10 mm	4.00	2.17, 7.37	
Margin			0.216
Well defined	Ref.		
Poorly defined	0.72	0.42, 1.21	
Aspect ratio	0.91	0.74, 1.27	0.817
Vascularity			
Type I	Ref.		
Type II	1.73	0.70, 4.26	0.235
Type III	1.24	0.74, 2.09	0.407
Type IV	0.62	0.27, 1.44	0.266
Calcification			
No	Ref.		
Microcalcification	1.49	0.91, 2.44	0.117
Macrocalcification	0.74	0.32, 1.71	0.483
Mixed mode	3.06	0.91, 10,29	0.070
CA			<0.001
No	Ref.		
Yes	2.53	1.50, 4.26	
Loss of echogenic capsule			0.819
No	Ref.		
Yes	1.10	0.50, 2.43	
SCLNM			0.014
No	Ref.		
Yes	2.50	1.20, 5.21	
Time to enhancement			
Late	0.78	0.22, 2.74	0.703
Simultaneous	1.14	0.35, 3.71	0.833
Enhancement intensity			
Hyper-enhancement	Ref.		
Iso-enhancement	0.64	0.19, 2.09	0.458
Hypo-enhancement	1.07	0.31, 3.69	0.916
Homogeneity of enhancement			0.536
Homogeneous	Ref.		
Heterogeneous	0.84	0.48, 1.46	
Discontinuous capsule enhancement			0.399
No	Ref.		
Yes	1.29	0.71, 2.33	

CA, capsule abutment; SCLNM, suspected cervical lymph node metastasis; OR, odds ratio; CI, confidence interval.

### Performance of the nomogram in the primary cohort and validation cohort

**Figure 3:**
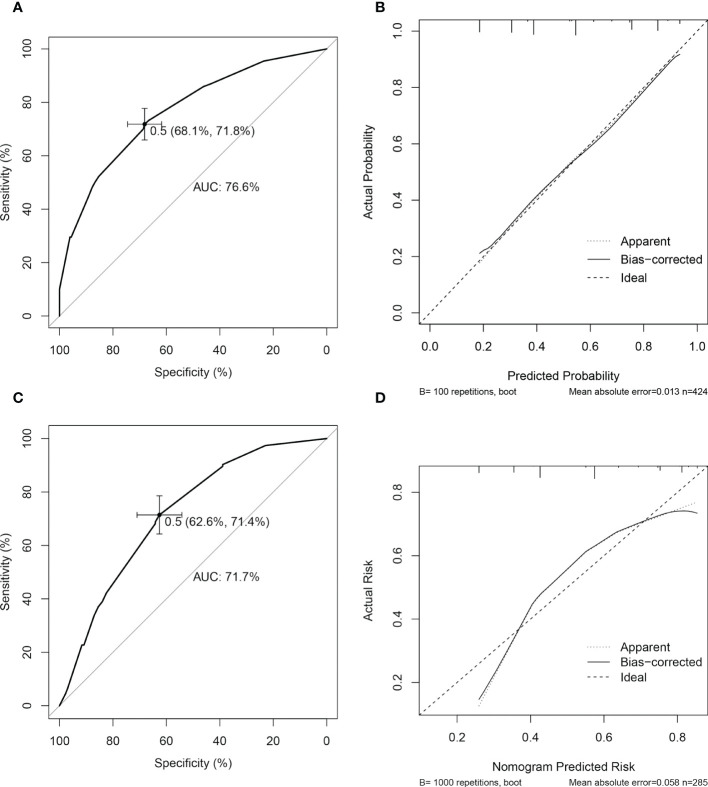
Performance of nomogram for predicting aggressiveness of papillary thyroid carcinoma. In the primary cohort, the performance of the predictive nomogram was evaluated by receiver operating curve analysis **(A)** and the calibration curve **(B)**. In validation cohort, the model was evaluated by the discrimination **(C)** and calibration **(D)** curves.

In ROC analysis, the optimum cutoff value was identified as 0.5. In the primary cohort, the AUC of the nomogram was 0.77 (95% CI, 0.72-0.81) and the sensitivity and specificity were 71.8% and 68.1%, respectively. The *Hosmer-*Lemeshow *test* showed good calibration (*P* = 0.967). The favorable discrimination and calibration were confirmed in the validation cohort, in which the AUC was 0.72 (95% CI, 0.66-0.78) and the sensitivity and specificity were 71.4% and 62.6%, respectively; the *Hosmer*–Lemeshow *test* yielded a *P* value of 0.251 (**Figure 3**).

### Inter-reader agreement of CUS and CEUS features

Margin, vascularity, CA, loss of echogenic capsule, SCLNM, time to enhancement, enhancement intensity, and discontinuous capsule enhancement showed substantial inter-reader agreement (*κ* coefficients = 0.774, 0.667, 0.773, 0.792, 0.697, 0.741, and 0.711, respectively), and shape, calcification and the homogeneity of enhancement showed almost perfect inter-reader agreement (*κ* coefficients =0.821, 0.907, and 0.878, respectively).

### Examples of using the nomogram to predict PTC aggressiveness

**Figure 4:**
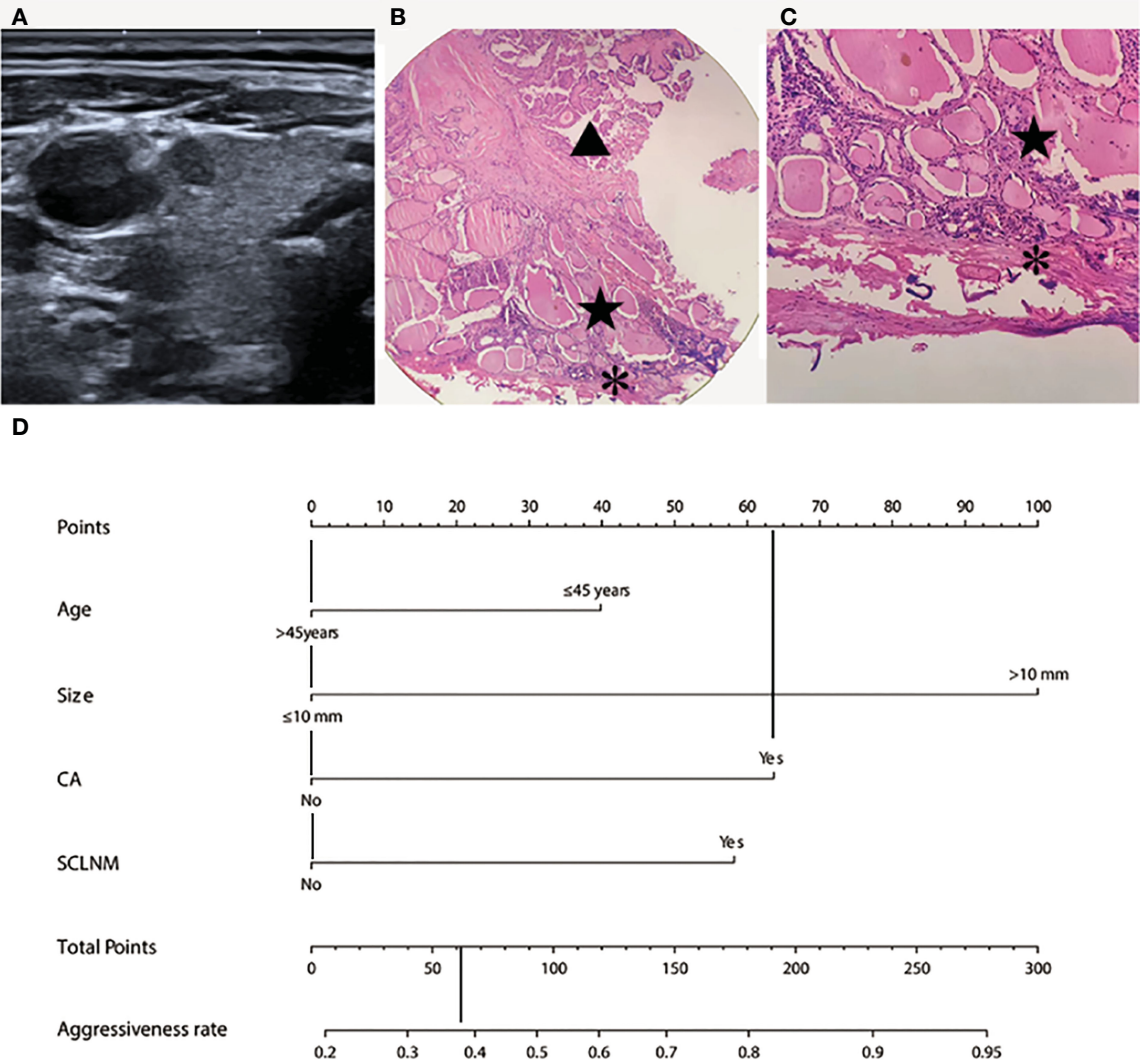
Example of nomogram usage for predicting non-aggressive papillary thyroid carcinoma. In patient 1 (male, 47 years old), preoperative fine needle aspiration cytology confirmed papillary thyroid carcinoma in the right middle part of the thyroid. **(A)** Conventional ultrasound showed the size was 4.3 mm, with capsule abutment (CA) and no suspected cervical lymph node metastasis (SCLNM). Pathological assessment revealed no capsular invasion. **(B)** and **(C)** showed normal thyroid tissue (pentagram) between papillary thyroid carcinoma (triangle) and the capsular (star) at different magnifications (HE staining, 100 ×and 200×, respectively). On the nomogram **(D)**, this patient showed a total score of approximately 62 and the predicted risk of aggressiveness was less than 0.4.

**Figure 5:**
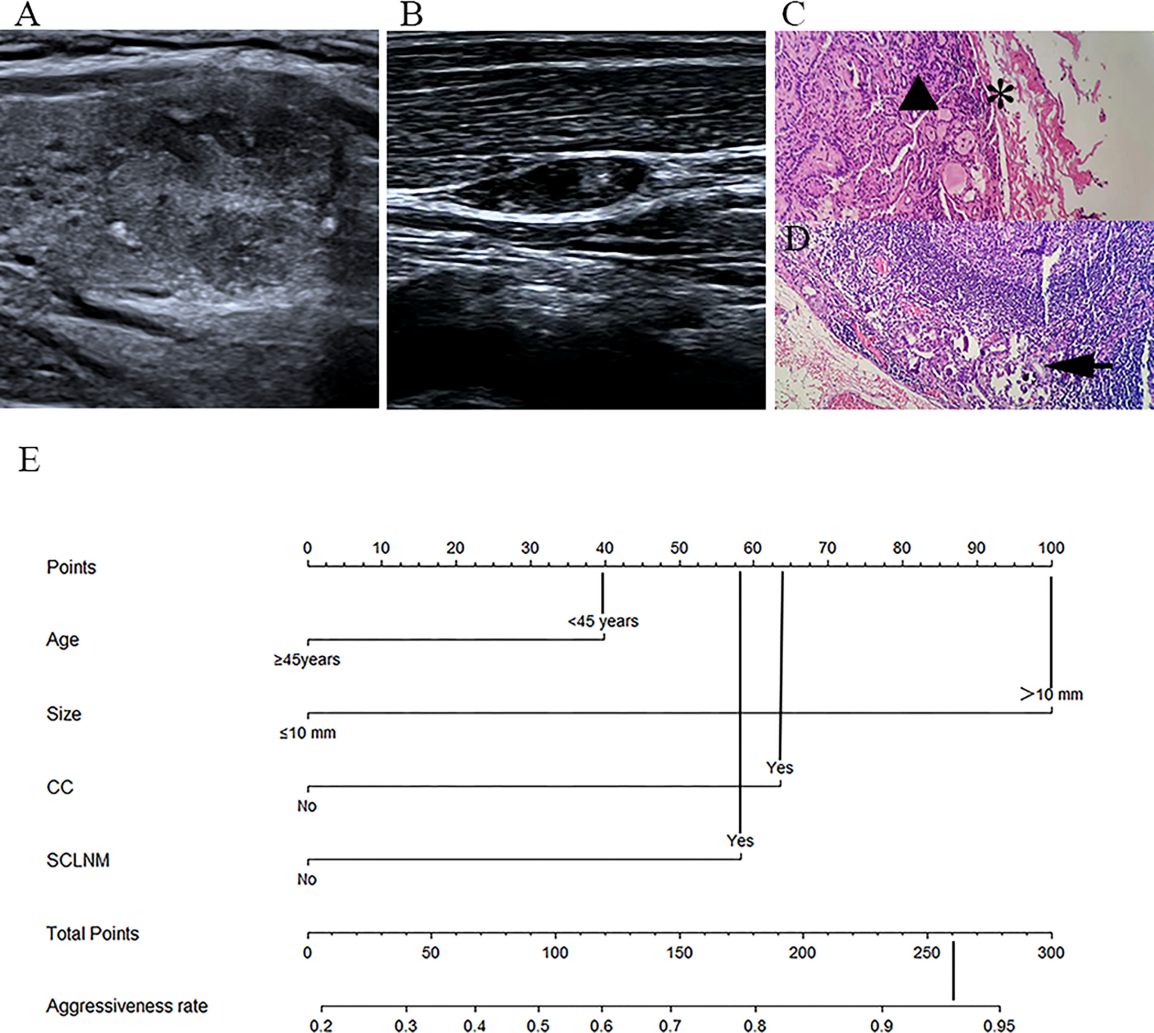
Example of nomogram usage for predicting aggressive papillary thyroid carcinoma. In patient 2 (female, 24 years old), preoperative fine needle aspiration cytology confirmed papillary thyroid carcinoma in the right lower part of the thyroid. **(A)** Conventional ultrasound showed that the tumor size was 17.8 mm, with capsule abutment (CA). **(B)** Conventional ultrasound on the right lateral neck level IV indicated suspected cervical lymph node metastasis (SCLNM) with loss of the fatty hilum and microcalcifications. Postoperative pathological assessments **(C, D)** revealed PTC (triangular) infiltrating the thyroid capsule (stellate) and metastasis (arrow) of the ipsilateral lateral neck lymph nodes (*HE* staining, 400×). On the nomogram **(E)**, the total score was approximately 260, and the predicted probability of aggressiveness was greater than 0.9.

The predicted risk of aggressiveness in patient 1 (age, >45 years; size, <10 mm, CA) was less than 0.4. Pathological assessments revealed a non-invasive PTC (**Figure 4**). For patient 2 (age, <45 years; size >10 mm, CA, SCLNM), the predicted probability of aggressive PTC using the nomogram was greater than 90%, and the tumor was confirmed as an aggressive PTC by pathology (**Figure 5**).

## Discussion

This study aimed to identify the factors associated with aggressiveness of PTC and to use these to construct a predictive nomogram for use in the clinic. We identified four variables—tumor size, patient age, CA, and SCLNM—that were independently associated with PTC aggressiveness. The nomogram constructed using these four variables showed good performance in external validation. Previous predictive models have included only one of the risk factors (e.g., ETE, CLNM) (27–30); our model, incorporating four predictive factors, will enable more accurate stratification of PTC according to aggressiveness. We tried to include CEUS indicators in this model; however, we found no significant differences in CEUS indices between non-aggressive and aggressive groups.

Correct evaluation of PTC aggressiveness is essential for treatment planning. Currently, ATA guidelines recommend active surveillance (AS) as an option in carefully selected patients, the aim being to avoid potential overtreatment while still identifying tumors that would continue to progress (11, 31). Reports from different countries have shown favorable outcomes with AS, but 1%-2% of patients will have progressive tumor growth and ETE (32–34). Therefore, more stringent selection of patients suitable for AS is needed. Stratification of PTC by aggressiveness is especially important for treatment selection in PTC patients with low recurrence risk. Current ATA guidelines recommend lateral lobectomy rather than total thyroidectomy for PTC patients with unifocal tumor of 1-4 cm size and no clinical evidence of lymph node metastasis and DM (11). However, the incidence of PTC recurrence in patients undergoing lobectomy is higher than that in patients undergoing total thyroidectomy, so some patients may have to undergo total thyroidectomy after the initial partial thyroidectomy (35). Thus, careful selection of the surgical approach is necessary for management of low-risk patients.

In this study, tumor size was the strongest independent predictor of PTC aggressiveness. Tumor size is an important prognostic indicator for PTC patients in clinical practice and is incorporated in the American Joint Committee on Cancer (AJCC) TNM staging system and other grading systems such as AGES, AMES, and MACIS. In the studies by de Castro et al. (36) and Sorrenti et al. (37), tumor size was associated with persistence and recurrence of PTC. In our study, tumors >10 mm were more likely to exhibit aggressive characteristics than tumors ≤10 mm. This is consistent with previous reports showing higher incidence of CLNM in patients with large PTC (>1 cm) than in patients with papillary microcarcinoma (38, 39). Lee et al. (40) reported that ETE is more commonly associated with larger PTCs (>10 mm) than with papillary microcarcinomas. Khan et al. (41) found that tumor size >2 cm was an independent risk factor for DM in patients with well-differentiated thyroid cancer. No patient in our study had DM, but our results confirmed that larger PTCs (>1 cm) were more likely to be aggressive. The results of this study suggest that clinicians could consider more conservative management for papillary thyroid microcarcinoma. This conclusion is consistent with the 2015 ATA recommendation of AS for “very low risk” PTC ≤1 cm in size.

CA was found to be an independent predictor of PTC aggressiveness in the present study. Intrathyroidal PTC could be considered to have a relatively lower risk of aggressive behavior. Seong et al. (42) reported that smaller distance between the PTC and the thyroid capsule is associated with higher incidence of CAI and ETE. Lee et al. (40) analyzed the degree of CA and found that increasing the CA ration could predict the ETE. In this study, we did not carry out quantitative analysis of CA parameters; we only defined CA as present or absent. This classification method helps improve the sensitivity of prediction of PTC invasiveness, because this can include those PTCs with only CAI on pathology. The results of this study suggest that PTC patients with CA should be managed more aggressively, while patients with PTC limited to the gland may be considered for more conservative treatment.

We found SCLNM on preoperative CUS to be another independent predictor of aggressiveness of PTC. PTC patients with SCLNM detected by preoperative ultrasound have greater risk of CLNM, which also implies greater risk of aggressiveness. Ultrasound examination of the neck can accurately identify lateral CLNM. Al-Hilli et al. (43) reported that ultrasound had sensitivity, positive predictive value, and accuracy of 78%, 89%, and 75%, respectively, for diagnosis of SCLNM. In addition, for PTC >2 cm in size, the accuracy of identification of multiple CLNM has been found to be as high as 89%. Wu et al. (44) established a machine-learning algorithm to predict central CLNM of PTC and found that SCLNM was an independent predictor of central CLNM. In another study, SCLNM was found to be associated with lymphatic vessel infiltration in the postoperative period (45). Thus, the results of this study suggest that SCLNM before surgery could be considered an indication for lateral neck lymph node resection in PTC patients.

Patient age was another independent predictor of PTC aggressiveness in our study. Aggressive PTC tended to be more common in patients aged <45 years than in patients >45 years. Our result was consistent with the report of Xu et al. (46). Tuttle et al. (33) also found that younger age at diagnosis was independently associated with the likelihood of tumor growth (hazard ratio per year, 0.92). Although PTC patients aged >45 years have less aggressive tumors, in the AJCC staging system for PTC, older age indicates a worse prognosis. This contradiction may be explained by the difference between the aggressiveness and patients’ outcome during tumor progression. In younger patients, the aggressive tumor may grow rapidly, but prognosis remains excellent even if metastasis occurs (47–49); meanwhile, in older PTC patients, less aggressive tumors will be associated with poor prognosis. The results of this study indicate that more aggressive management should be applied in young patients (< 45 years).

This study attempted to construct a nomogram to predict the risk of invasiveness of solitary PTC, providing a new method for clinicians dealing with solitary PTC and may provide a reference for screening appropriate patients for AS. In fact, multiple PTCs are very common in clinical practice, and multifocality in thyroid cancer is a significant risk factor for disease progression and increases the risk of disease recurrence. Patients with multiple PTC are more likely to have ETE and CLNM (50, 51), and the use of AS for multiple PTC is still controversial (52, 53). DM of PTC is uncommon, but is associated with PTC aggressiveness, and DM in thyroid cancer significantly reduces survival in PTC patients (41, 54, 55). On the basis of the guidelines of the American Thyroid Association, DM-PTC is considered to indicate a high risk and usually requires more aggressive treatments (11). Hence, more studies involving DM-PTCs should be carried out in the future. In this study, no statistically significant difference was found in the distribution of Hashimoto thyroiditis between the aggressive and non-aggressive groups. This result is consistent with Cappellacci et al. (56), who found that, although Hashimoto thyroiditis was an independent predictor of differentiated thyroid cancer, the distribution of Hashimoto thyroiditis was similar in patient groups with different aggressive features (e.g., gross ETE, microscopic ETE, vascular invasion, aggressive PTC variants, and extensive CLNM).

This study has several limitations. First, this was a single-center study with a small sample size; our results need to be confirmed in large multi-center studies. Second, no patient had DM in our study; the clinical and ultrasound characteristics of these rare cases need further study. Third, this study only considered solitary PTCs; the findings may not be applicable in patients with multiple PTCs.

## Conclusion

The nomogram constructed using these four preoperative indices appears to reliably predict the risk of aggressiveness of solitary PTC. The nomogram might be helpful for management strategy selection in patients with PTC.

## Data availability statement

The raw data supporting the conclusions of this article will be made available by the authors, without undue reservation.

## Ethics statement

The studies involving human participants were reviewed and approved by The ethics committee of Shanghai General Hospital. Written informed consent for participation was not required for this study in accordance with the national legislation and the institutional requirements.

## Author contributions

LL and RW conceived the idea and designed the whole study. LL, CJ, GL participated in the data collection and image analysis. LL performed the statistical analysis. LL participated in manuscript preparation. QS, LD participated in methodology support. All authors contributed to the article and approved the submitted version.
